# Effect of spherical Au nanoparticles on nanofriction and wear reduction in dry and liquid environments

**DOI:** 10.3762/bjnano.3.85

**Published:** 2012-11-15

**Authors:** Dave Maharaj, Bharat Bhushan

**Affiliations:** 1 Nanoprobe Laboratory for Bio- & Nanotechnology and Biomimetics (NLBB), The Ohio State University, 201 W. 19th Avenue, Columbus, Ohio 43210-1142, USA

**Keywords:** AFM, drug delivery, friction, gold nanoparticles, MEMS/NEMS, nanomanipulation

## Abstract

Nano-object additives are used in tribological applications as well as in various applications in liquids requiring controlled manipulation and targeting. On the macroscale, nanoparticles in solids and liquids have been shown to reduce friction and wear. On the nanoscale, atomic force microscopy (AFM) studies have been performed in single- and multiple-nanoparticle contact, in dry environments, to characterize friction forces and wear. However, limited studies in submerged liquid environments have been performed and further studies are needed. In this paper, spherical Au nanoparticles were studied for their effect on friction and wear under dry conditions and submerged in water. In single-nanoparticle contact, individual nanoparticles, deposited on silicon, were manipulated with a sharp tip and the friction force was determined. Multiple-nanoparticle contact sliding experiments were performed on nanoparticle-coated silicon with a glass sphere. Wear tests were performed on the nanoscale with AFM as well as on the macroscale by using a ball-on-flat tribometer to relate friction and wear reduction on the nanoscale and macroscale. Results indicate that the addition of Au nanoparticles reduces friction and wear.

## Introduction

Nano-objects are continually studied in tribological applications and increasingly in other applications that require controlled manipulation and targeting in liquid environments. The need for suitable forms of lubrication for micro/nanoelectromechanical systems (MEMS/NEMS) and the ability to control and transport nano-objects in liquids, requires an understanding of nano-object behavior, with regards to friction, adhesion and wear, which is essential to their successful and continued application.

Increasing the lifetime and efficiency of individual components of systems is crucial to the commercialization of MEMS/NEMS [1]. As one moves from the macroscale to the micro/nanoscale, surface to volume ratio increases. Therefore, adhesive and friction forces, which are dependent on surface area, become more significant. With MEMS/NEMS devices, the initial start-up forces and torques needed become high, which can hinder device operation and reliability [2]. The choice of a suitable lubricant on these scales becomes crucial.

**Figure 1:**
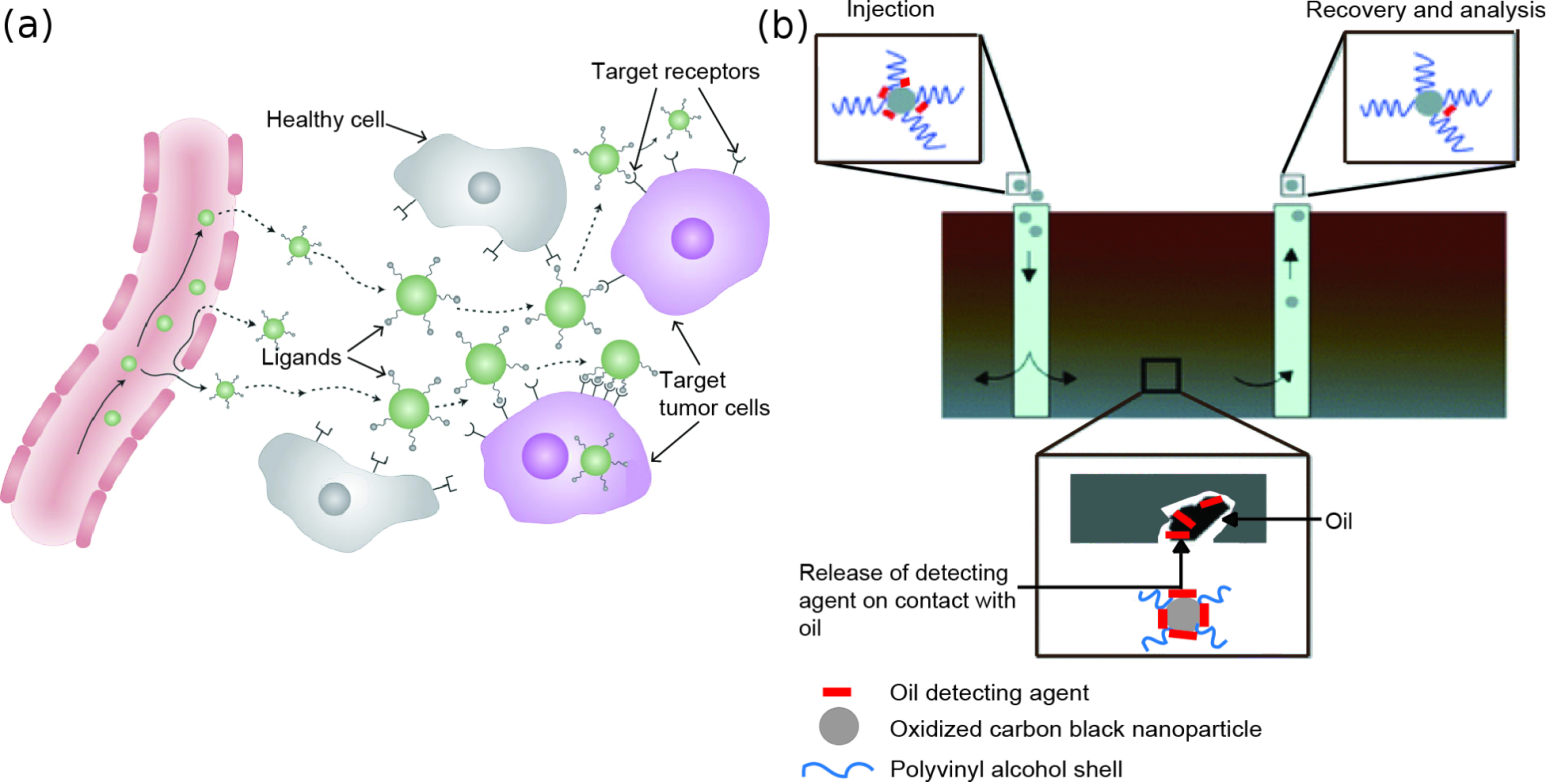
(a) Schematic of drug-carrying nanoparticles targeting cancer cells and releasing their therapeutic payload resulting in death of the cancer cell. Reprinted by permission from Macmillan Publishers Ltd [8], copyright 2011. (b) Showing the process of oil detection with nanoparticles. The carbon-black nanoparticles are coated with an oil-detecting agent. After injection into the ground, the agent is released on contact with hydrocarbons and this is used as an indication of the presence of oil on recovery and analysis of the nanoparticle [10]. Reproduced by permission of the Royal Society of Chemistry.

Nano-objects are also used for applications that require controlled manipulation and targeting mechanisms in biomedicine and the oil industry. Applications include, but are not limited to, their use in targeted drug delivery and chemical sensors in the identification of oil, removal of contaminants and enhanced oil recovery (EOR). Au, iron oxide, polymer and silica nanoparticles have been studied in targeted drug delivery [3–8]. In cancer treatment, nanoparticles are either functionalized with biomolecules that recognize and attach to the cancer cells, [6–7] or in the case of iron-oxide nanoparticles, the nanoparticles are directed by an external magnetic field [9]. The cells are destroyed by drugs that coat the nanoparticles or by increasing the temperature of the nanoparticles to which cancer cells are susceptible. Figure 1a shows a nanoparticle loaded with a therapeutic drug and functionalized with a biomolecule (ligand), which selectively attaches to receptors in the cancer cell. The drug is then released as the nanoparticle diffuses into the diseased cell resulting in cell death.

Several factors need to be considered for the successful use of nanoparticles in targeted drug delivery. Biological barriers, including physical surfaces and the reticulo-endothelial system (RES), which detects and sequesters blood-borne particles, can prevent nanoparticles from reaching their intended target [7]. Smaller nanoparticles can diffuse through surfaces and avoid detection by the RES. Studies have shown that forces such as hydrodynamic and van der Waals forces along with the nanoparticle size influence lateral drift (margination) and adhesion to cell walls [5,11], which are important factors for effective drug delivery.

In oil-detection studies, as in the example shown in Figure 1b, oxidized carbon-black nanoparticles with a polyvinyl alcohol shell are coated with an oil-detecting agent (2,2’,5,5’-tetrachlorobiphenyl (PCB)). The release of this agent on contact with hydrocarbons is used as an indication of the presence of oil on recovery of the nanoparticles [10]. In contaminant removal, nanocomposites composed of collagen and superparamagnetic iron-oxide nanoparticles (SPIONs) have been investigated. The collagen selectively absorbs the oil by motion of the nanoparticles towards the oil in a magnetic field [12]. Magnetic nanoparticles are also of interest in enhanced oil recovery (EOR) since they can be dispersed in fluid and manipulated and monitored by an external magnetic field [13–14]. In both oil detection and EOR, agglomeration of nanoparticles can prevent flow through porous media. Nanoparticles can adhere to the surface over which they flow, which results in losses and prevents their eventual recovery [12,15]. Studies have shown that surface charge can cause nanoparticles in liquids to adhere to sites in porous media and hinder mobility [15]. Functionalizing nanoparticles with a hydrophilic polymer has been shown to reduce aggregation and improve flow [12].

For many of these applications, control of the friction of nanoparticles moving in the fluids, as well as the friction and adhesion as nano-objects come into contact with each other and surfaces present in their working environment, is necessary.

Nano-object additives have proven to be successful in macroscale studies in reducing friction and wear when added to solid materials and base-liquid lubricants and are expected to provide similar benefits on the micro/nanoscale. Some examples of nano-objects in liquids and their reported sizes, for friction and wear reduction, with studies carried out on the macroscale, are as follows: WS_2_ platelets (0.5 µm) in commercial mineral oil [16], ferric oxide nanoparticles (20–50 nm) in 500 solvent neutral (SN) mineral oil [17], spherical MoS_2_ (15–60 nm) in poly-alpha-olefin (PAO) and 150 SN [18], spherical WS_2_ nanoparticles (50–350 nm) in SN 150 and SN 190 [19], spheroidal carbon-nano-onion nanoparticles (<10 nm) in PAO [20], WS_2_ nanoparticles (120 nm) in paraffin oil [21], MoS_2_ spheres (0.5–3 µm) in 500 SN oil [22] and carbon spheres (420 nm) in water [23]. Mechanisms for friction and wear reduction have been reported as tribofilm formation, rolling, sliding, and reduced contact area. It is expected that the reduced contact area and mobility offered by nano-objects observed on the macroscale will also lead to friction reduction and wear protection on the micro/nanoscale. These micro/nanoscale contacts are relevant for MEMS/NEMS devices.

In MEMS/NEMS devices, commercial lubricant oils are unacceptable as base liquids on machine components running in liquid. This is due to energy losses associated with the large viscous drag. In experiments where electrostatic micromotors are operated in a liquid environment, there have been problems of excessive drag and damping, which limited operating speeds, due to the use of high viscosity (20–60 cSt) oils [24]. However, studies have also demonstrated that friction and wear can be reduced with liquids of low viscosities [25]. Liquids such as glycerol and dodecane have been shown to reduce friction and wear. Glycerol has a dynamic viscosity (934 mPa·s) that is significantly higher than water (0.89 mPa·s) and studies were performed on the macroscale by using pin-on-disk testers [26]. In these studies, glycerol was also combined with water to lower the viscosity, which may be feasible for micro/nanoscale applications. Dodecane has been used as a base fluid with ZnS nanorod additives [27], which also resulted in a reduction in the coefficient of friction and wear. Tests were performed by using a surface force apparatus (SFA) with crossed-mica geometry with a 0–1600 µm^2^ contact area.

To characterize friction forces associated with controlled manipulation and to understand the nature of the mechanism of friction and wear reduction of nanoparticles in MEMS/NEMS devices, studies have been carried out in both single-nanoparticle contact and multiple-nanoparticle contact with the aid of an AFM. Both mechanisms are described in detail in the following section.

### Single-nanoparticle contact

**Figure 2:**
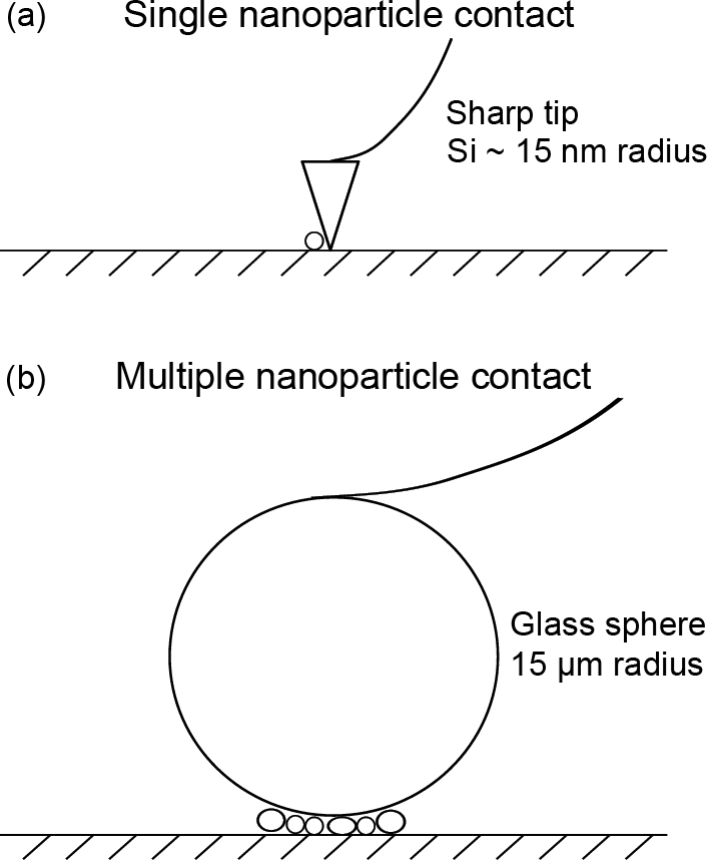
Schematics of (a) a sharp tip pushing a particle in single-particle contact and (b) a glass sphere sliding over several particles in multiple-particle contact.

In single-nanoparticle contact, a sharp AFM tip, as shown in Figure 2a as an example, is used to push the nanoparticle laterally (lateral manipulation). Manipulation studies of nanoparticles, with the aid of an AFM have shown that there is a contact-area dependence of the friction force. Several types of nanoparticles with reported diameters, such as latex spheres (80–100 nm) [28], Sb nanoparticles (120–400 nm) [29], (50–500 nm) [30], spherical SiO_2_ nanoparticles (30 nm) [31] and spherical Au nanoparticles (25 nm) [32], (30–50 nm) [31] and (80 nm) [33] have been studied in both contact and intermittent-contact modes in dry environments. In liquid environments, Au nanoparticles (20–30 nm) have also been manipulated in water and ethanol with an AFM operated in intermittent-contact mode [34]. In addition to the contact-area dependence of friction observed in these studies, the relative-humidity (RH) dependence of friction was investigated by Mougin et al. [32] and Palacio and Bhushan [31]. In the study by Mougin and co-workers [32], it was found that Au nanoparticles could not be moved in an ultrahigh vacuum (UHV) as compared to an ambient environment under otherwise identical manipulating conditions. Palacio and Bhushan [31] found that for larger nanoparticles, the friction force was lower at lower RH (10%) compared to higher RH (40%) for both Au and SiO_2_ particles. Both studies were performed on silicon substrates. This would suggest that some adsorbed moisture between the nanoparticle and substrate is necessary for enhanced lubricity.

Manipulation studies of nanoparticles submerged in liquid environments, to simulate nanoscale contacts and characterize friction forces, are limited. Such studies are necessary for simulating the kinds of environments that involve controlled-manipulation and targeting-mechanism applications of nanoparticles. In addition, these studies provide insights into the interactions of single nanoparticles with a surface, in dry and submerged-in-liquid environments.

### Multiple-nanoparticle contact

In addition to determining the friction force due to lateral manipulation, the effect of the normal load on the friction force has also been investigated. In multiple-nanoparticle contact, a glass sphere attached to an AFM cantilever, as shown in Figure 2b as an example, was used to slide over several nanoparticles. This type of study simulates the contacts experienced by MEMS/NEMS devices when nanoparticles are introduced for the purpose of friction and wear reduction.

Previous studies have been performed using a colloidal glass sphere attached to an AFM cantilever on bare silicon surfaces [35] and in multiple-nanoparticle contact with both immobile asperities on polymer surfaces [36] and mobile nanoparticles, such as spherical Au and SiO_2_ nanoparticles on silicon surfaces [31]. In these studies, friction forces were reduced due to the reduced contact area provided and, in the case of Au and SiO_2_, the possible sliding and possible rolling of individual nanoparticles. Similar to single-nanoparticle contact studies, AFM studies of multiple-nanoparticle contacts submerged in a liquid environment are also lacking. These studies are crucial to determine the added advantage of dispersing nanoparticles in liquids, in cases where the entire MEMS/NEMS system is submerged in a liquid environment. This has the ability to eliminate the adhesive effects of meniscus forces associated with the formation of capillary bridges due to adsorbed moisture on a surface.

### Objective of this research

In this study, spherical Au nanoparticles are investigated to determine their effect on friction and wear under dry conditions and submerged in water. Lateral manipulation of single nanoparticles with a sharp tip is used to determine the friction force between the nanoparticle and the silicon substrate by AFM. The coefficient of friction is also investigated, with the aid of a glass sphere attached to an AFM cantilever sliding over multiple nanoparticles. Wear tests were performed on the nanoscale by using AFM and on the macroscale by using a ball-on-flat tribometer. This helps to link the nanoscale friction and wear to that observed on the macroscale and to fully understand the mechanisms involve.

## Experimental

### Choice of nanoparticle and operating liquid

As mentioned previously, Au is attractive for use in biomedicine since it is a noble metal, does not oxidize readily and has low to no toxic effects [6]. This also makes it suitable for use as a solid lubricant, and studies in liquids on the nanoscale to determine friction reduction and wear protection have not been reported. Spherical Au nanoparticles have a well-characterized shape and this thus eliminates orientation effects of tubular or cylindrical nanoparticles on the observed friction forces. Additionally, its small contact area and mobility is expected to contribute to friction force reduction and, when added to a base liquid, to further reduce the coefficient of friction and wear. Au nanoparticles are also suitable for manipulation studies since they are found in applications requiring controlled manipulation and targeting.

Liquids such as glycerol and dodecane have been shown to reduce friction and wear, as previously mentioned. However, our attempts to combine glycerol with Au nanoparticles suspended in deionized (DI) water lead to agglomeration of the nanoparticles. Evidence of agglomeration was observed by a change in color of the solution, from red to purple. In the case of dodecane, its immiscibility with water prevented its use with Au nanoparticles suspended in DI water.

In addition to the fact that Au nanoparticles are provided already suspended in DI water, the low viscosity of water and its ability to provide a surface of low shear strength [35] for sliding, makes it a good candidate as an operating fluid. Water is also attractive due to its environmentally friendly nature.

### Materials and sample preparation

**Figure 3:**
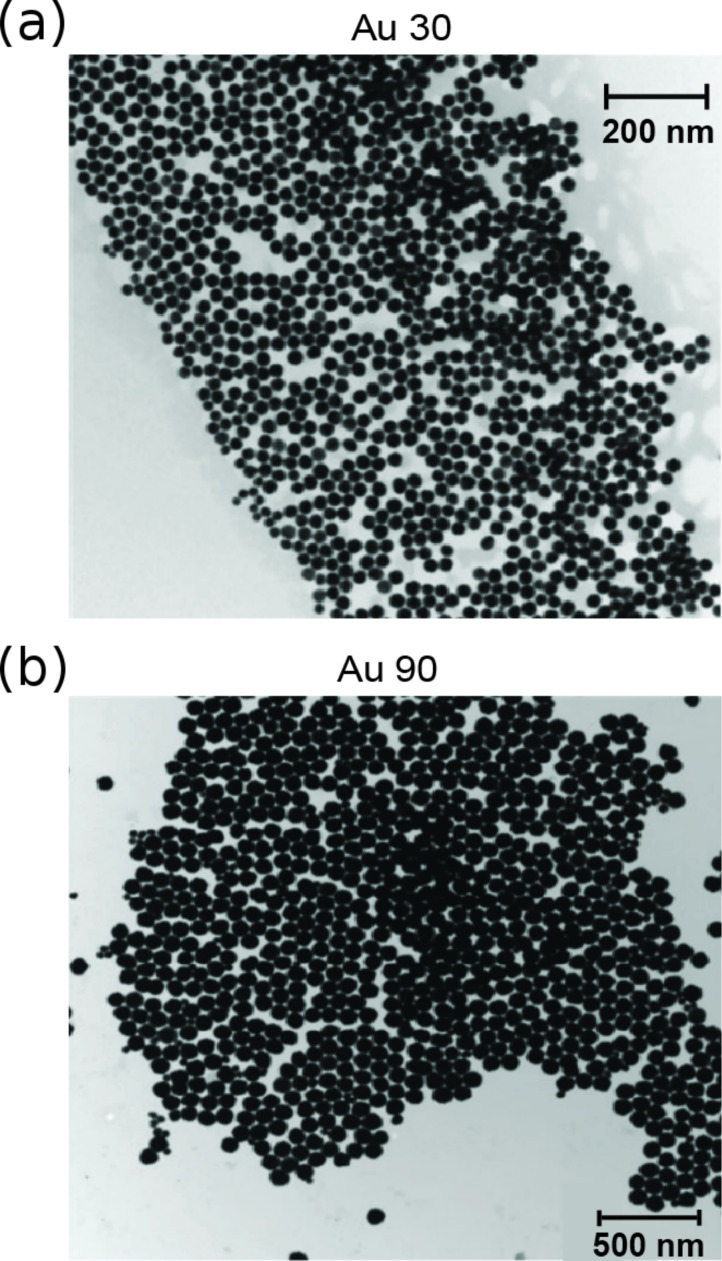
TEM images of spherical Au nanoparticles approximately (a) 30 nm in diameter and (b) 90 nm in diameter [37].

Si (100) silicon wafers with a native oxide layer (University Wafers, Boston, MA) were ultrasonically cleaned in DI water, followed by isopropyl alcohol (IPA) and finally acetone for 15 min each. For experiments involving nanoparticle-coated surfaces under dry conditions, several droplets of Au nanoparticles suspended in DI water (Nanopartz, Inc., Loveland, CO) were deposited onto the clean Si (100) substrate by using a syringe. A 25% concentration of an initial 0.05 mg/mL solution was used for all sliding and wear experiments unless otherwise stated. The substrate was then placed on a hot plate and heated to a temperature of about 70–80 °C and left until the water evaporated. The nominal diameters for the Au nanoparticles as reported by the manufacturer were about 30 and 90 nm, to be henceforth referred to as Au 30 and Au 90, respectively. Figure 3 shows transmission electron microscopy (TEM) images of spherical Au 30 and Au 90 nanoparticles [37].

**Figure 4:**
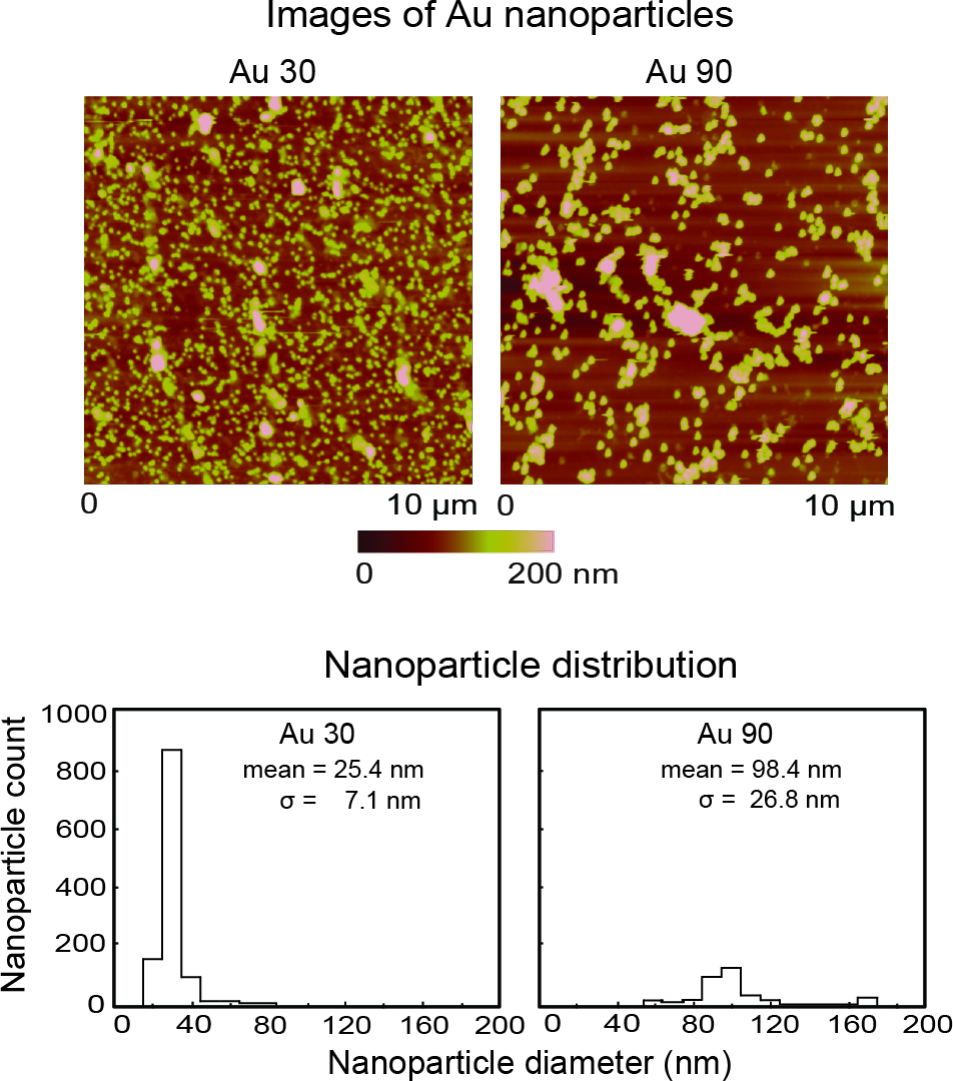
Topography map of Au 30 and Au 90 with corresponding histograms depicting the nanoparticle size distribution. The average nanoparticle diameter for Au 30 is 25.4 ± 7.1 nm and for Au 90 is 98.4 ± 27 nm from each histogram.

Typical nanoparticle distributions obtained from topography images by using a commercial AFM (Multimode, Bruker, Santa Barbara, CA) are shown in Figure 4, for a 10 µm × 10 µm scan size. The average nanoparticle diameter for Au 30 is 25.4 ± 7.1 nm and for Au 90 is 98.4 ± 27 nm. The nanoparticle coverage analysis was performed by using SPIP 5.1.11 (Image Metrology A/S, Horshølm, Denmark). Imaging was performed at a normal load of 10 nN.

For experiments on nanoparticles submerged in water, a fluid cell consisting of a standard multimode cantilever holder (Bruker, Santa Barbara, CA) with a glass plate glued just above the cantilever was placed over the silicon substrate. The resulting meniscus bridge formed between the glass plate and substrate completely encloses the cantilever, which eliminates any viscous effects and adhesion due to meniscus forces.

### Nanoscale friction force

Friction force data for nanoscale experiments were obtained by using the previously mentioned AFM for both single- and multiple-nanoparticle contact. The friction signals obtained in both cases were converted to forces by using an established calibration method [2,38]. Normal loads were determined by multiplying the cantilever vertical deflection by the cantilever stiffness [2]. The vertical deflection in turn was obtained by operating the cantilever in force-calibration mode, in which the deflection sensitivity obtained from the force curve was multiplied by the change in setpoint voltage.

For single-nanoparticle contact, a sharp silicon tip (FORT series, Applied NanoStructures, Inc., Santa Clara, CA,) with a spring constant *k* = 3 N/m and nominal radius of 15 nm was used for manipulation of a single Au nanoparticle under dry conditions. For submerged-in-water conditions, a silicon nitride tip of lower force constant was used (Orc8 series, Bruker, Camarillo, CA) with *k* = 0.05 N/m and a nominal radius of 15 nm. For dry conditions, a 10% concentration of Au nanoparticles was used to ensure that isolated single nanoparticles could be found and would not be hindered by other nanoparticles during a manipulation attempt. The average value of the friction force presented is the result of five manipulations.

In multiple-nanoparticle contact, to determine the coefficient of friction, a soda lime glass sphere (Duke Scientific Corporation, Palo Alto, CA) of nominal radius 15 µm attached to a silicon probe (FORT series, Applied NanoStructures, Inc., Santa Clara, CA,) with a spring constant *k* = 3 N/m was used. Coefficient of friction data were obtained by plotting the friction force as a function of normal load from five random spots on the test samples.

### Nanoscale wear

Wear tests on the nanoscale were performed by using the glass sphere attached to a silicide coated cantilever (NANOSENSORS, Neuchatel, Switzerland) with *k* = 40 N/m. In this case a cantilever of higher stiffness was used to obtain a normal load of 20 µN, which is not possible with the cantilevers mentioned previously. This was carried out for 1, 10 and 100 cycles at 10 Hz, over a 5 µm × 5 µm scan size. A larger scan (10 µm × 10 µm) was then taken of the area enclosing the wear region for comparison. Tests were not performed under submerged-in-water conditions, since at lower cycles, as the test is completed, nanoparticles suspended in solution will continue to be deposited on the surface as the water evaporates, and hence cover the wear area. Representative data for 5–6 tests are summarized in the results section. All experiments were performed at room temperature (23 °C) and 50–55% relative humidity.

### Macroscale friction and wear

For comparison to the nanoscale, macroscale friction tests were conducted by using a ball-on-flat tribometer to determine if similar effects would be observed on both scales. For these tests, Au 90 was chosen as a representative nanoparticle. A sapphire ball of 1.5 mm radius was fixed to a stationary holder. A normal load of 200 mN was applied to the surface of the substrate and the tribometer was operated in a reciprocating manner [25]. The stroke length was 10 mm with an average speed of 3.5 mm/s. Friction forces were measured with semiconductor strain gages for 500 cycles. In liquid environments, 2–3 droplets of DI water with and without Au 90 nanoparticles were deposited onto the silicon substrate with a syringe. The sapphire ball was then slid over the substrate. The coefficient of friction was obtained as a function of the number of cycles. Wear was characterized by using an optical microscope by taking micrographs of the wear scars created during the test.

## Results and Discussion

In this section, results for experiments in single- and multiple-nanoparticle contact are given for dry conditions and submerged-in-water conditions. In single nanoparticle contact, the manipulation technique is first described for each condition, and then the friction forces for both Au 30 and Au 90 nanoparticles are compared and discussed for both dry conditions and submerged in water. For multiple-nanoparticle contact, friction forces and corresponding coefficient of friction data are given and explained in detail. In addition, wear data for both nanoscale wear in dry conditions and macroscale wear in dry and submerged-in-water conditions, with and without the addition of Au nanoparticles are also presented. AFM wear images are shown for nanoscale wear. For macroscale wear, optical and SEM micrographs and corresponding data for the coefficient of friction are given and discussed.

### Single-nanoparticle contact: Lateral manipulation of nanoparticles over a silicon substrate

#### Manipulation technique in dry and liquid environments

**Figure 5:**
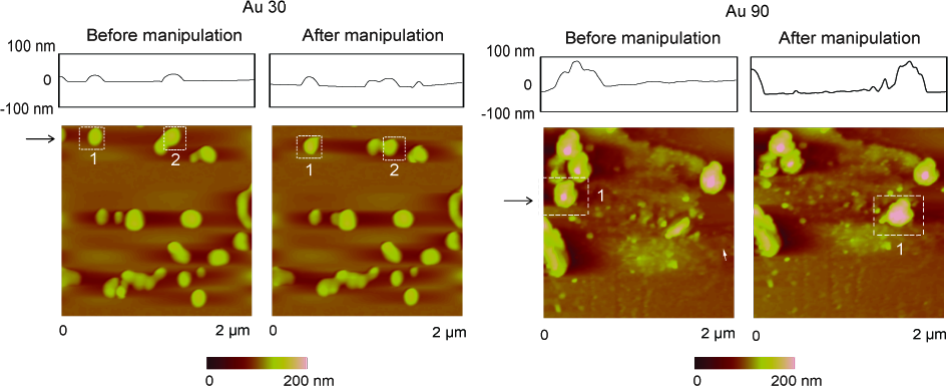
Two examples of topography maps and height profiles, at sections shown by the arrows, of Au 30 and Au 90 nanoparticles highlighted by the squares, manipulated within the same scan area. For Au 30, nanoparticles 1 and 2 are moved, and for Au 90, nanoparticle 1 is moved.

For single-nanoparticle contact under dry conditions, a sharp tip is used to push Au nanoparticles in the lateral direction. Figure 5 shows examples of topography images of nanoparticles, highlighted by the squares before and after manipulation within the same scan area, for both Au 30 and Au 90. A normal load of 10 nN was used during imaging. This takes place on the scan line illustrated by the black arrows. A 2 µm × 2 µm scan area is used to ensure that the nanoparticle can still be seen in the same image after manipulation. This clearly shows that the nanoparticles are being moved by the AFM tip. The smaller Au 30 nanoparticles indicated by 1 and 2 are pushed a shorter distance as the tip stays in contact for a shorter time compared to a larger Au 90 nanoparticle (1). This occurs since the Au 90 nanoparticles have a larger radius and take longer to roll or slide out of contact with the tip.

**Figure 6:**
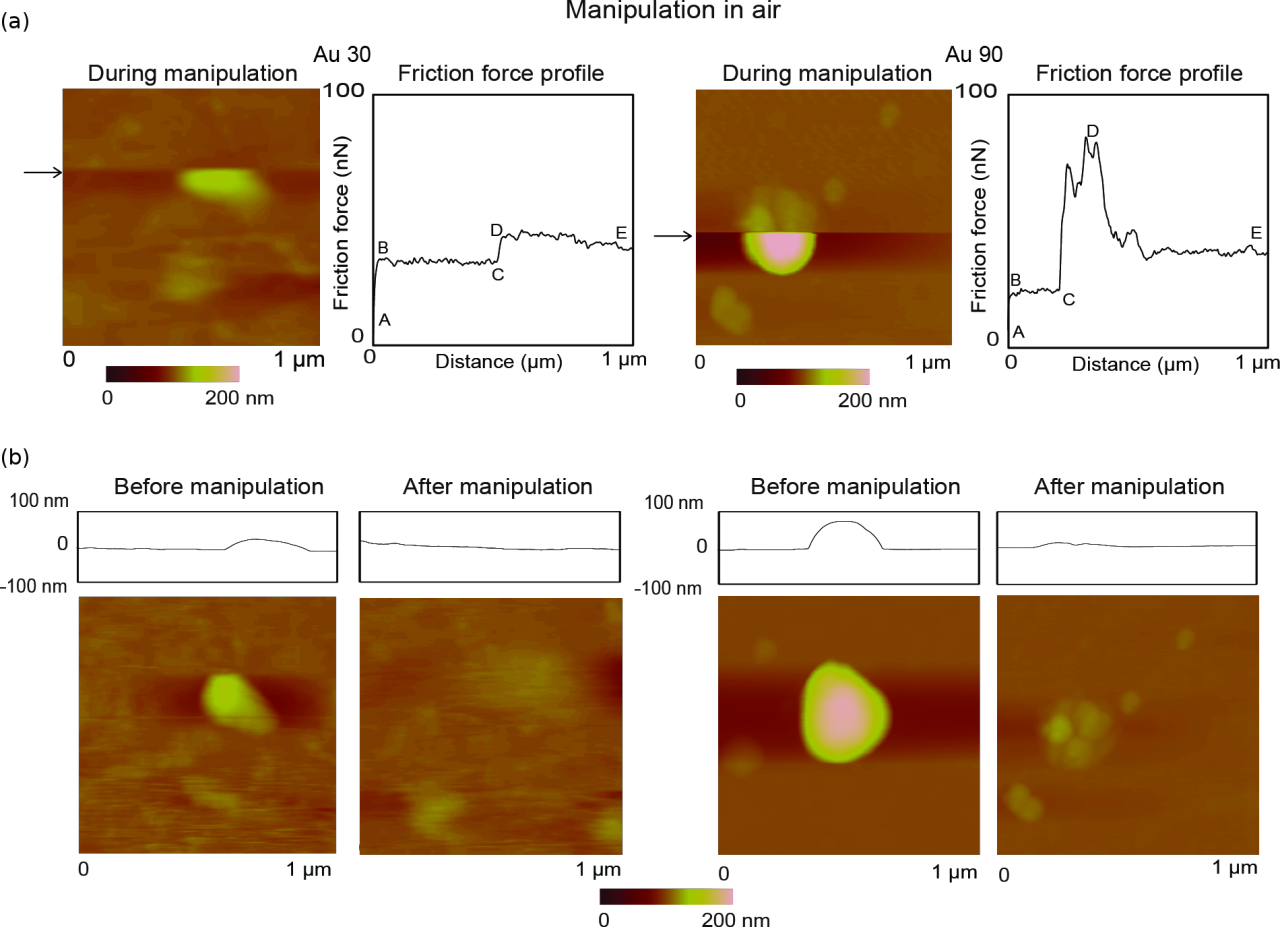
(a) Topography maps and 2-D friction force profiles of Au (30 nm in diameter) and Au (90 nm in diameter) during manipulation of single nanoparticles in dry conditions. During manipulation, the normal load is increased (A–B) at the approximate center of the particle image (indicated by the black arrows) from 10 nN to 300 nN on a single scan line. On this scan line, the sharp tip slides laterally (B–C) until contact is made with the particle. The lateral friction force (C–D) increases, as the sharp tip pushes against the particle, until it begins to slide. E shows the end of the scan line. (b) Two examples of topography images and height profiles showing the same scan area before and after manipulation.

Figure 6 shows examples of topography maps and 2-D friction force profiles for Au 30 and Au 90 nanoparticles. A 1 µm × 1 µm area is imaged before manipulation to identify nanoparticles of interest. During manipulation in the same scan area, as shown in Figure 6a, scanning proceeds in the slow scan direction (bottom to top), and the normal load is increased from 10 nN to 300 nN at the approximate center of the nanoparticle. This corresponds to an increase in the friction signal (A–B) on a single scan line, as illustrated by the black horizontal arrows. Increasing the normal load prevents the nanoparticle from being imaged, as the cantilever tip remains in contact with the substrate and does not slide over the nanoparticle to track its height. As the sharp tip continues to slide along the scan line from left to right (fast-scan direction), there is a further increase in the friction signal (C–D), which directly correlates to the twisting of the cantilever as it pushes against the nanoparticle, until friction is overcome and sliding begins. Point E represents the end of the scan line on which the manipulation takes place.

Corresponding profile and topography images of the same scan region before and after nanoparticle manipulation are shown in Figure 6b, which provide proof that the nanoparticle is pushed out of the scan area. Imaging of the nanoparticle was done in contact mode for both pre- and postmanipulation. The change in lateral force (C–D) is used to quantify the friction force between the nanoparticle and silicon surface as sliding is initiated.

**Figure 7:**
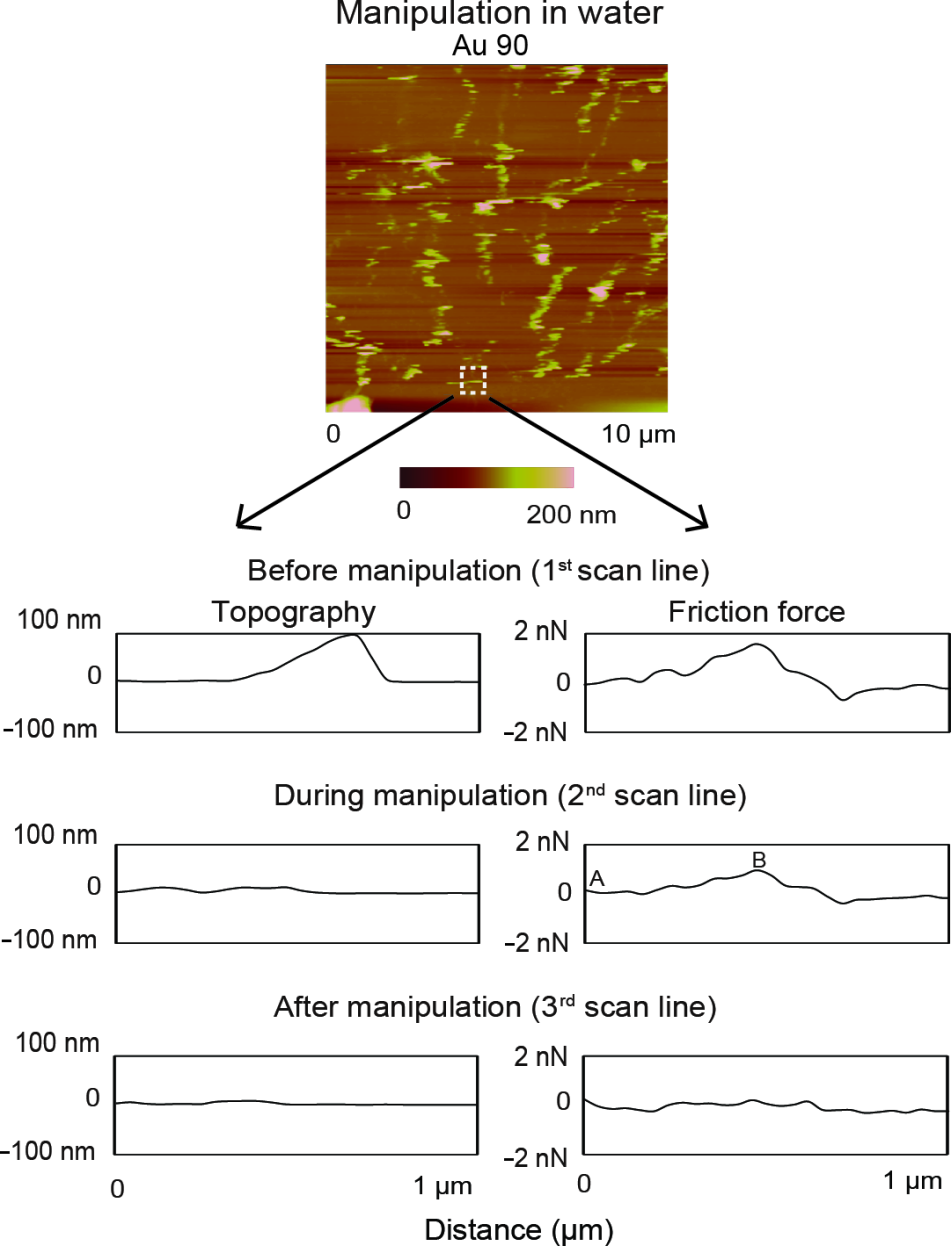
Topography map of Au (90 nm in diameter) nanoparticle submerged in water. Imaging is performed by using a cantilever with a low normal load of 1 nN. The highlighted particle is examined and the associated topography and friction force scan lines before manipulation, during manipulation and after manipulation are show. Before manipulation there is a rise in friction force corresponding to the particle profile as the cantilever deflects during imaging. During manipulation the topography profile is flat as the nanoparticle is being pushed, which corresponds to a rise in friction force (A–B). After manipulation, both the topography and friction force profiles remain flat as the nanoparticle is no longer there.

For submerged-in-water conditions as shown in Figure 7, a 10 µm × 10 µm area is imaged at a normal load of 1 nN. This allows for multiple manipulations within a single topography scan. As an example, a single partial image of a nanoparticle, highlighted by the white squares, is used to demonstrate the manipulation technique. The associated topography and friction-force scan lines, before manipulation (Figure 7, top), during manipulation (Figure 7, middle) and after manipulation (Figure 7, bottom), for the above-mentioned nanoparticle are also shown. In this case the normal load does not have to be increased since the nanoparticles can be pushed due to the low adhesion between them and the substrate during scanning. As the nanoparticle is imaged, there is a rise in friction force associated with twisting of the cantilever (Figure 7, top) corresponding to the nanoparticle profile. In the middle set of scan lines, the topography is flat since the tip no longer follows the nanoparticle profile as it is being pushed, which corresponds to an increase in friction force. This increase (A–B) corresponds to the friction force between the Au nanoparticle and the silicon substrate at the initiation of sliding. In the bottom set of scan lines, both the topography and friction-force scan lines remain flat, which proves that the nanoparticle has been pushed out of the area. The friction-force results for nanoparticle manipulation under dry conditions and submerged in liquid is presented and discussed in the following section.

#### Comparison of friction forces obtained during lateral manipulation under dry and submerged- in-water conditions

**Figure 8:**
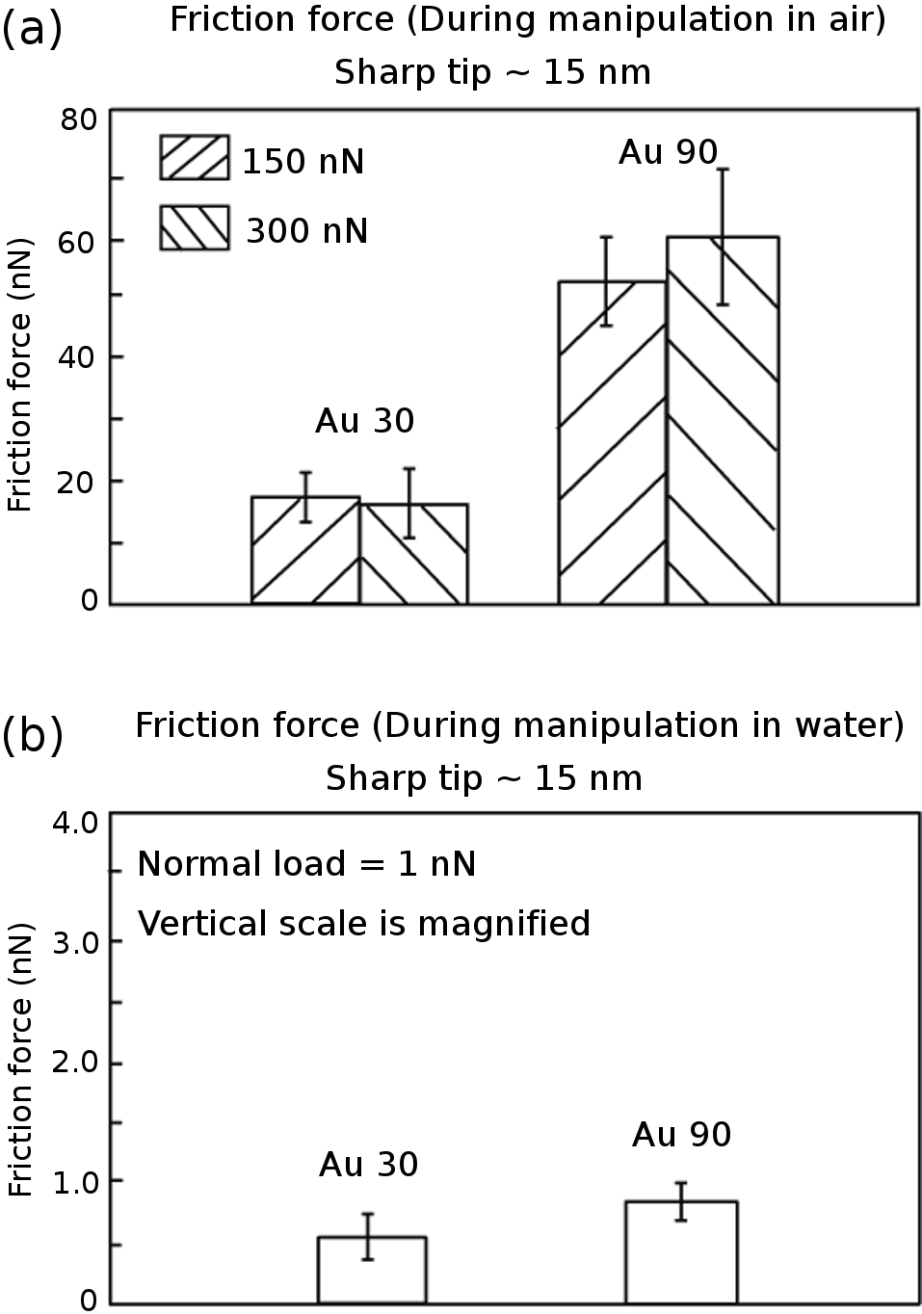
Friction force for Au 30 and Au 90 nanoparticles on the silicon substrate during manipulation, at normal loads of (a) 150 nN and 300 nN in air and (b) 1 nN in water. The vertical scale has been magnified for data in water.

Figure 8 shows the friction forces during nanoparticle manipulation of Au 30 and Au 90 nanoparticles under dry conditions and submerged in water. The data shows that Au 90 exhibit higher friction forces compared to Au 30. The normal load acting on the nanoparticle is due only to the mass of the nanoparticle since it is pushed from the side and the friction force is the result of adhesion between the nanoparticle and the silicon substrate. The adhesive force can include van der Waals forces under both dry and submerged-in-water conditions and meniscus forces under dry conditions. In this regime the friction force is not proportional to the normal load since it is dependent on the contact area. The friction force in this case, for single-nanoparticle contact of spherical shapes is proportional to (normal load)^2/3^ [1–2,31,39]. The normal load comprises the external normal load and the adhesive force. Since the adhesive force is dependent on surface area, it is expected that the larger Au 90 nanoparticles will display higher changes in friction force compared to the smaller Au 30 nanoparticles, and this is confirmed from the results shown in Figure 8a–b for both dry and submerged-in-water conditions.

In addition to comparing the friction force for Au 30 and Au 90 in dry conditions, Figure 8a also compares the friction force when the normal loads are 150 nN and 300 nN. The friction forces are comparable as is expected, since the normal load should only influence the interaction between the tip and the substrate. The friction force between the Au nanoparticle and the substrate provides the additional offset C–D in the friction signal shown in Figure 6a during manipulation in air, independent of the tip–substrate friction force [30]. If the tip–substrate friction force approaches that of the nanoparticle–substrate force, it is expected that the friction signal during manipulation would be masked. This would necessitate lowering of the normal load as in the case of nanoparticles submerged in water.

Figure 8b presents the results of measurements of the average friction force for the Au 30 and Au 90 nanoparticles submerged in water. Since the high normal loads used during manipulation under dry conditions would mask the friction-force signal, a lower normal load of 1 nN is used for nanoparticle manipulation. This is sufficient since the nanoparticles are weakly adhered to the substrate and can be easily moved during the manipulation process shown in Figure 7. The adhesive forces are due to van der Waals interactions since there are no meniscus bridges formed under the submerged-in-water conditions. The lower friction forces observed under the submerged-in-water conditions compared to the dry conditions can thus be attributed to the nanoparticles sliding on an easily sheared surface and the elimination of meniscus forces.

### Multiple-nanoparticle contact-sliding of a glass sphere over several nanoparticles under dry and submerged-in-water conditions

#### Nanoscale friction

**Figure 9:**
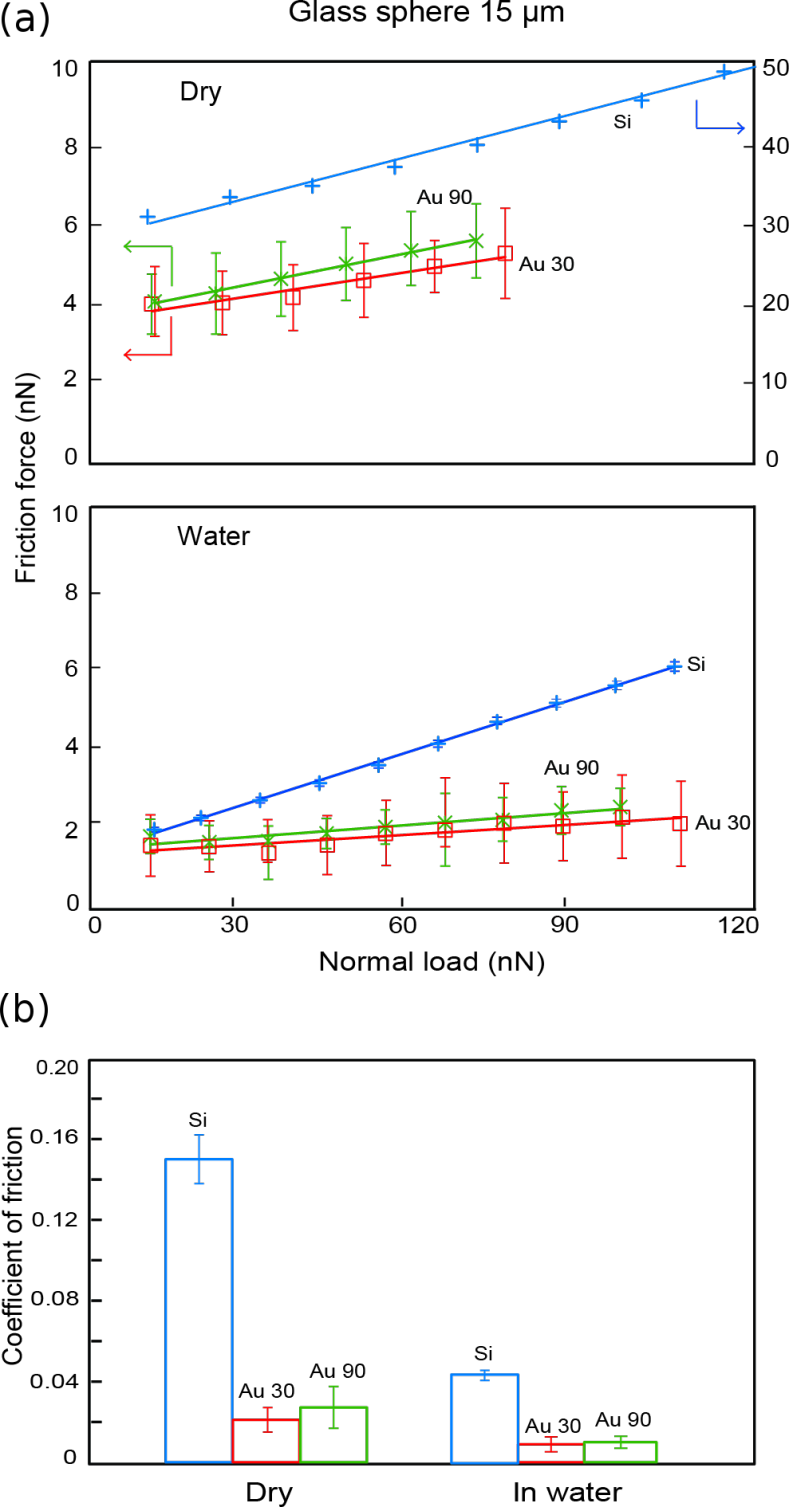
(a) Friction force as a function of normal load and (b) coefficients of friction for both dry and in-water conditions, with and without Au nanoparticles.

In multiple-nanoparticle contact, the effect of the normal load acting on the Au nanoparticles between two surfaces is studied to determine the effects on the friction force. Figure 9 summarizes the friction forces and coefficient of friction under dry and submerged-in-water conditions. In general, the friction forces were lower for sliding in water as compared to sliding under dry conditions, as shown in Figure 9a, for both nanoparticle-coated and uncoated surfaces. The same trend is observed in the data for the coefficient of friction (Figure 9b). Sliding in multiple-nanoparticle contact results in lower coefficients of friction under dry and submerged-in-water conditions, as compared to sliding on the silicon substrate. The coefficient of friction is also lower for sliding on Au 30 nanoparticles compared to Au 90. This is expected since the lateral manipulation of the nanoparticles resulted in lower friction forces for Au 30 nanoparticles compared to Au 90. The difference is more pronounced under the dry conditions compared to sliding in water. One reason for this could be that, under the submerged conditions, since the nanoparticles and cantilever are completely covered by water, the meniscus force contribution to the friction force is eliminated. One must also consider that, since the glass sphere is glued to the cantilever, the addition of the epoxy could contribute to an increased stiffness *k* of the cantilever, making it less sensitive to detecting changes in the lateral friction-force signal, especially for sliding in water where friction-force signals are lower. In the case of multiple-nanoparticle contact with an applied external load, the friction force shows a linear relationship as evidenced by the results for the coefficient of friction in Figure 9b.

It has also been demonstrated that sliding on multiple asperities on nanopatterned surfaces [36] results in the reduction of friction. In this particular case, the asperities are immobile, and reduction occurs as a result of the reduced contact area. For sliding on Au nanoparticles, friction-force reduction can be attributed to the mobility of the nanoparticles in addition to the reduced contact area. It is expected that as the glass sphere comes into contact with the Au nanoparticles, some of them will be deformed, since the larger nanoparticles will be encountered first and experience the highest contact pressures, due to fewer particles supporting the normal load. The resulting friction-reduction mechanism can thus be attributed to the reduced contact area, the sliding over deformed nanoparticles, and individual nanoparticles sliding with the glass sphere. In addition, it is also possible for some rolling to take place as the sphere encounters a greater number of nanoparticles and the contact pressure is reduced, leading to undeformed nanoparticles, which may roll between the surfaces.

In water, the presence of a liquid film between the glass sphere and the silicon substrate provides an interface of low shear strength resulting in a lower coefficient of friction [35]. In addition, since the glass sphere, cantilever and Au nanoparticles are completely covered in water, meniscus forces are eliminated, which also contributes to the reduction in the friction force.

#### Nanoscale wear

**Figure 10:**
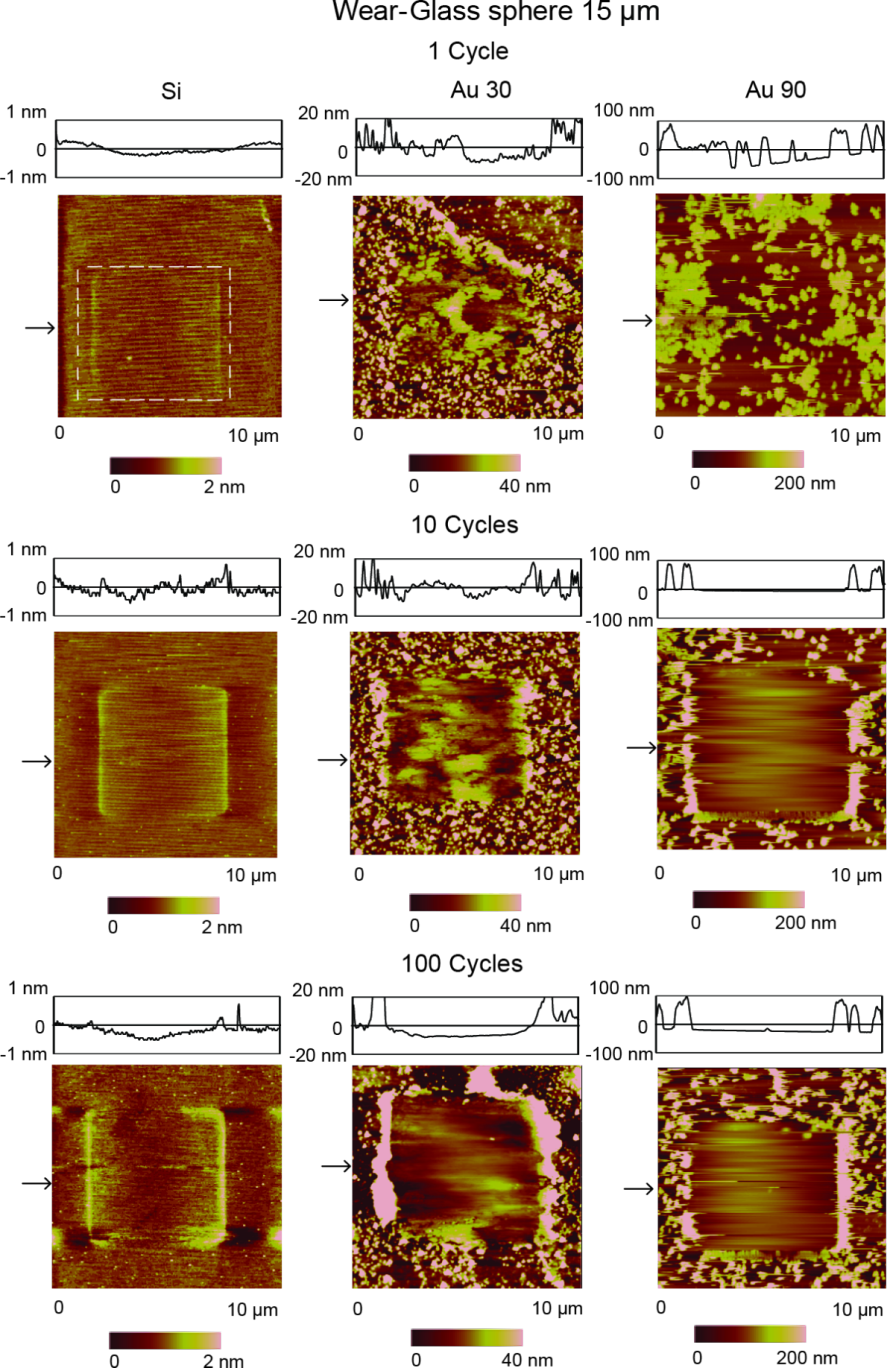
Topography maps and 2-D profiles, at sections shown by the arrows, after sliding for 1, 10 and 100 cycles with a normal load of 20 µN on Si and Si coated with Au 30 and Au 90.

For a potential lubricant to be considered effective, it must not only be able to reduce the coefficient of friction, but also protect the underlying surface. Figure 10 summarizes the wear data for sliding on Si, and Si coated with Au 30 and Au 90 for 1, 10, and 100 cycles under dry conditions. As sliding progresses, a greater degree of wear is observed for the uncoated silicon substrate for 10 cycles compared to 1 cycle, and 100 cycles compared to 10 cycles, as seen within the first column. As we move from 1 to 10 cycles there is some roughening of the surface evidenced by the height profile. After 100 cycles a small amount of material has been removed, with a wear depth close to 0.5 nm. The very small amount of material removed at a load of 20 µN after 100 cycles would indicate that the wear mechanism is most likely due to breaking and removal of sharp asperities, as seen in adhesive wear [25], and eventual polishing of the surface as evidenced by the smoother height profile as the number of cycles progresses from 10 to 100.

For the surface coated with Au 30, as the number of cycles progresses from 1 to 10, it can be observed that nanoparticles still remain in the wear area, with evidence from the height profiles suggesting that they become compressed and deformed. At 100 cycles the nanoparticles are completely removed from the wear area and show agglomeration on the edges. For the surface coated with Au 90, after 1 cycle the nanoparticles are just beginning to be pushed out of the area and are completely removed after 10 cycles, in contrast to the surface coated with Au 30. This can be explained by the number of nanoparticles typically found on the surface. From the coverage data displayed in Figure 4, it can be seen that there is a much higher nanoparticle count for the Au 30 nanoparticles compared to Au 90 nanoparticles. It is expected that it would therefore take a longer time (more cycles) to completely remove the Au 30 nanoparticles from the surface.

It is also expected, since the softer Au nanoparticles remain in the wear area after 1 cycle for Au 90 and 10 cycles for Au 30, that the damage to the silicon surface should be less than that of an initially uncoated substrate, since the glass sphere is not directly sliding on the underlying surface and the contact load being exerted contributes towards deformation of the Au nanoparticles. Additionally, since Au 30 nanoparticles remain in the wear area longer than Au 90 nanoparticles, less wear of the surface is expected. Adhesive and abrasive wear of the silicon substrate is thus minimized since the asperities of the softer Au nanoparticles are more likely to deform and fracture during sliding than are those of the substrate or the glass sphere. At 100 cycles it is therefore expected that there would be less wear for the Au 30 surface compared to Au 90, with the greatest wear occurring on the bare silicon substrate.

#### Macroscale friction and wear

**Figure 11:**
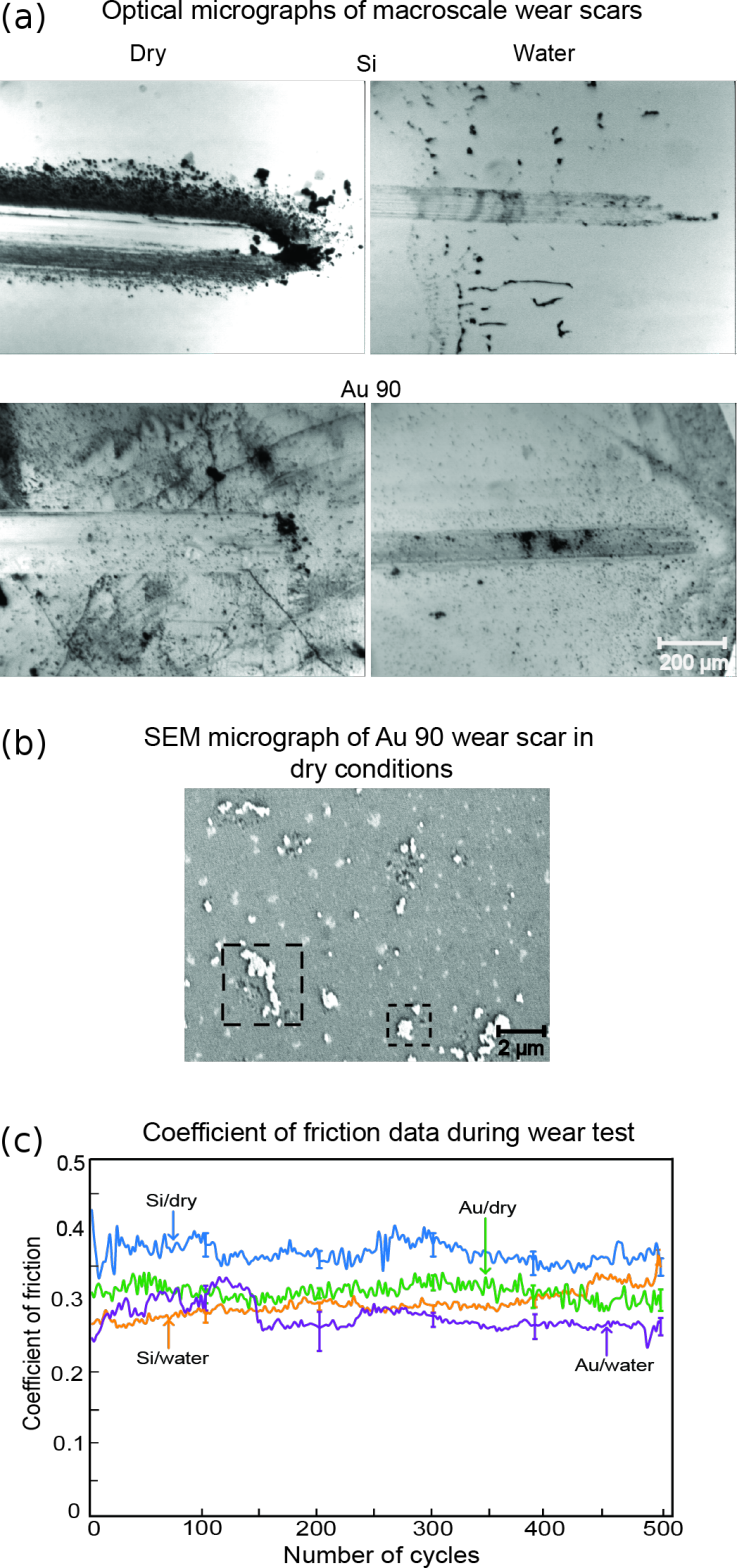
(a) Optical micrographs of the wear scars taken after 500 cycles. (b) SEM micrograph of the wear scar for agglomerated Au 90 nanoparticles, within the squares, under dry conditions. (c) Coefficients of friction from ball-on-flat tests, for both dry and water conditions, with and without Au 90 nanoparticles.

The results of the ball-on-flat wear tests are shown in Figure 11. Optical micrograph images of the wear scars for dry and water conditions with and without Au 90 nanoparticles are displayed in Figure 11a. In general, the widths of the wear scars shown are larger for sliding under dry conditions compared to sliding under water conditions, as the amount of wear is greater. Under dry conditions, the addition of Au 90 nanoparticles reduces the amount of wear compared to the uncoated silicon substrate. Under water conditions, the widths of the wear scars are comparable with or without the addition of nanoparticles.

Figure 11b shows a magnified scanning electron microscope (SEM) micrograph of the Au 90 wear scar under dry conditions, where agglomerations of Au 90 nanoparticles can be seen, highlighted by the squares. This is in contrast to Figure 3b, which shows TEM images of single unagglomerated nanoparticles. Agglomeration occurs during the wear process as nanoparticles are pressed together. The presence of the Au 90 nanoparticles within the wear scar contributes to the reduction in the coefficient of friction and wear by reducing the contact area, sliding and rolling of the nanoparticles.

Figure 11c shows data of the coefficient of friction for the four wear cases over 500 cycles. Higher coefficients of friction occur under dry conditions compared to water conditions. The lowest coefficients of friction coincide with the cases of least wear observed in Figure 11a. The results are similar to those on the nanoscale, where the submerged-in-water conditions generally produce lower coefficients of friction than those under the dry conditions, with the lowest coefficients of friction being observed with Au nanoparticles as shown in Figure 9b.

The addition of Au nanoparticles creates a barrier between the two surfaces and reduces the contact area. Similar to the nanoscale friction, it is expected that the observed reduction in the coefficient of friction is due to sliding on deformed nanoparticles, where deformation can occur when the initial contact is made with larger nanoparticles (fewer in number) resulting in higher contact pressure. It is also expected that some nanoparticles slide along with the ball as the number of cycles increases. As more nanoparticles support the load the contact pressure is reduced. This increases the chances of rolling for the undeformed nanoparticles as part of the friction-reduction mechanism.

In the case of DI water without Au nanoparticles, a surface of low shear strength is obtained, which makes it easier for the sapphire ball to slide [35]. Eventually, as the number of cycles continues, the coefficient of friction increases as wear of the substrate begins to take place and progresses. With the addition of Au 90 nanoparticles in DI water, the coefficient of friction is initially high and becomes lower after an initial settling-in period, which coincides with the initial deformation of the larger nanoparticles and eventual formation of a surface of low shear strength for sliding. In addition, as sliding progresses, the Au nanoparticles are continually being deposited on the surface, which replenishes the supply of nanoparticles for the sapphire ball to slide on. This combined with the low shear strength of the water contributes to the lowest observed coefficient of friction.

## Conclusion

An investigation of the effects of spherical Au nanoparticles on friction and wear reduction was carried out. Both single- and multiple-nanoparticle contact cases were studied by using an AFM for nanoscale studies. For macroscale studies, a ball-on-flat tribometer was used.

For single-nanoparticle contact, there is a friction-force dependence on the size of the nanoparticle with lower forces observed under submerged-in-water conditions. For multiple-nanoparticle contact, sliding over Au nanoparticles in general reduced the coefficient of friction as compared to sliding on the bare silicon substrate. Coefficients of friction were also lower under submerged-in-water conditions compared to dry conditions for all surfaces due to the low shear strength of the surface provided.

In nanoscale wear experiments, addition of the Au nanoparticles provides protection from wear of the underlying substrate by preventing the glass sphere from coming directly into contact with the surface. Evidence of nanoparticle deformation was found in the case of the Au 30 nanoparticles. A larger nanoparticle count was also responsible for the better wear protection afforded by the Au 30 nanoparticles compared to the Au 90 nanoparticles.

Macroscale studies using a ball-on-flat tribometer showed similar trends to those on the nanoscale. The addition of Au 90 nanoparticles under dry conditions and suspended in water resulted in lower coefficients of friction. The addition of Au 90 nanoparticles also resulted in better wear resistance in both cases, with the best wear protection and lowest coefficients of friction being observed in water.

From the results obtained, Au nanoparticles prove to be a good potential lubricant as it lowers the coefficient of friction and minimizes wear. Further studies with other nano-objects under dry conditions and as an additive to water or other low-viscosity liquids could open up the possibilities for new types of hybrid lubricants. Such lubricants are expected to contribute to the increased lifetime and efficiency of MEMS/NEMS devices, which will lead to their successful commercialization. In addition, the study of manipulation of new types of nanoparticles in different liquids will lead to an understanding of their suitability for various applications in which friction forces are of concern in controlled manipulation and targeting mechanisms.
